# Evaluating cost per remission and cost of serious adverse events of advanced therapies for ulcerative colitis

**DOI:** 10.1186/s12876-022-02590-6

**Published:** 2022-12-06

**Authors:** Vipul Jairath, Russell D. Cohen, Edward V. Loftus, Ninfa Candela, Karen Lasch, Bob G. Schultz

**Affiliations:** 1 Western University Schulich School of Medicine, London, ON Canada; 2 University of Chicago Pritzker School of Medicine, Chicago, IL USA; 3 Mayo Clinic College of Medicine and Science, Rochester, MN USA; 4 Takeda Pharmaceuticals U.S.A., Inc., 95 Hayden Ave., Lexington, MA 02421 USA; 5 University of Illinois at Chicago College of Pharmacy, Chicago, IL USA

**Keywords:** Biological products, Cost-effectiveness, Drug safety, Remission, Ulcerative colitis

## Abstract

**Background:**

Determining the relative cost-effectiveness between advanced therapeutic options for ulcerative colitis (UC) may optimize resource utilization. We evaluated total cost per response, cost per remission, and cost of safety events for patients with moderately-to-severely active UC after 52 weeks of treatment with advanced therapies at standard dosing.

**Methods:**

An analytic model was developed to estimate costs from the US healthcare system perspective associated with achieving efficacy outcomes and managing safety outcomes for advanced therapies approved for the treatment of UC. Numbers needed to treat (NNT) for response and remission, and numbers needed to harm (NNH) for serious adverse events (SAEs) and serious infections (SIs) were derived from a network meta-analysis of pivotal trials. NNT for induction and maintenance were combined with drug regimen costs to calculate cost per clinical remission. Cost of managing AEs was calculated using NNH for safety outcomes and published costs of treating respective AEs.

**Results:**

Costs per remission were $205,240, $249,417, $267,463, $365,050, $579,622, $750,200, and $787,998 for tofacitinib 10 mg, tofacitinib 5 mg, infliximab, vedolizumab, golimumab, adalimumab, and ustekinumab, respectively. Incremental costs of SAEs and SIs collectively were $136,390, $90,333, $31,888, $31,061, $20,049, $12,059, and $0 for tofacitinib 5 mg, golimumab, adalimumab, tofacitinib 10 mg, infliximab, ustekinumab, and vedolizumab (reference), respectively.

**Conclusions:**

Tofacitinib was associated with the lowest cost per response and cost per remission, while vedolizumab had the lowest costs related to SAEs and SIs. Balancing efficacy versus safety is important when evaluating the costs associated with treatment of moderate-to-severe UC.

**Supplementary Information:**

The online version contains supplementary material available at 10.1186/s12876-022-02590-6.

## Background

Inflammatory bowel disease (IBD), ulcerative colitis (UC), and Crohn’s disease have a prevalence of approximately 0.5% in the United States (US) [1]. Containing costs in UC has become challenging due to the considerable burden of disease. It has been estimated that the current population of patients with UC will cost approximately $377 billion to the healthcare system over their lifetimes, collectively [2]. The average lifetime incremental cost for each patient with UC is $230,000, but can be as much as $370,000 for those diagnosed when they are children [2]. Of note, the greatest costs of care (> $25,000 per patient) are incurred in the first year following diagnosis and are driven predominantly by treatment costs and complications from relapsing disease that result in emergency department visits and hospitalizations [3].

Advanced therapies, such as biologics and small molecules, are associated with high drug costs compared with conventional therapies [4, 5]. Yet advanced therapies have demonstrated higher cost-effectiveness compared with conventional therapies due to improved efficacy and subsequent avoidance of UC-related complications, such as surgery [6–8]. However, the ability to compare cost-effectiveness of advanced treatments has been less clear due to limitations in comparative evidence [6–8]. A recently published network meta-analysis included both indirect and direct treatment comparisons for advanced therapies in UC, allowing for the development of stronger comparative research, including cost-effectiveness [5]. Safety profiles between advanced therapies were also compared in the network meta-analysis, allowing for inclusion of costs on serious adverse events (SAEs) to be included in cost-effectiveness research [5].

This study aimed to evaluate the cost per response and cost per remission of advanced therapies for patients with moderately-to-severely active UC over 52 weeks, while also evaluating the costs of SAEs and serious infections (SIs).

## Methods

### Model structure

An analytic model was developed to estimate cost per response, cost per remission, and costs associated with managing SAEs and SIs for advanced therapies approved for the treatment of UC in the US. The comparators included intravenous (IV) vedolizumab [9], IV infliximab [10], oral tofacitinib (2 different maintenance regimens) [11], subcutaneous (SC) adalimumab [12], SC ustekinumab [13], and SC golimumab [14]. Induction regimens for the comparators were IV vedolizumab 300 mg at 0, 2, and 6 weeks, IV infliximab 5 mg/kg at 0, 2, and 6 weeks, oral tofacitinib 10 mg twice daily for at least 8 weeks, SC adalimumab 40 mg (4 injections in 1 day or 2 injections per day for 2 consecutive days), IV ustekinumab single dose using weight-based dosing (260 mg for up to 55 kg, 390 mg for > 55 kg to 85 kg, 520 mg for > 85 kg), and SC golimumab 200 mg at week 0, followed by 100 mg at 2 weeks. Maintenance regimens for the comparators were IV vedolizumab 300 mg every 8 weeks (Q8W), IV infliximab 5 mg/kg Q8W, oral tofacitinib 5 or 10 mg twice daily, SC adalimumab 40 mg every 2 weeks, SC ustekinumab 90 mg Q8W, and SC golimumab 100 mg every 4 weeks. The base case was defined as the drug acquisition cost and reimbursable cost (as determined from the Medicare limit file) for infusion/hour for drugs that are infused, and did not include added costs for administration services. The base case analysis assumed the comparators were administered according to their respective FDA-approved dosing regimens. The population contains a mixture of those who are anti–tumor necrosis factor (TNF) naïve and anti-TNF experienced, reflective of patients from the pivotal trials [5]. Cost per response, cost per remission, and costs on the management of SAEs and SIs were estimated over a 52-week time period. Induction and maintenance periods were calculated respectively and then combined.

### Model inputs

Comparative efficacy and safety inputs were derived from a previously published network meta-analysis of pivotal trials [5]. Numbers needed to treat (NNT) for response and remission from the network meta-analysis were used to estimate drug costs associated with achieving the respective outcomes at 52 weeks. Clinical response was defined based on complete Mayo scores (which assess stool frequency, rectal bleeding, physician’s global assessment, and endoscopic findings) and partial Mayo scores (which omit the endoscopic findings). Except for VARSITY, all studies defined clinical response as a reduction in complete Mayo score of ≥ 3 points and ≥ 30% from baseline with an accompanying decrease in rectal bleeding subscore of ≥ 1 point or absolute rectal bleeding subscore of ≤ 1 point, and clinical remission as a complete Mayo score of ≤ 2 points and no individual subscore > 1 point. In VARSITY, endoscopy was not performed at week 6. Therefore, for the purpose of these analyses, clinical response in VARSITY was defined as a partial Mayo score reduction of ≥ 2 points and ≥ 25% from baseline with an accompanying decrease in rectal bleeding subscore of ≥ 1 point or absolute rectal bleeding subscore of ≤ 1 point, and remission as a partial Mayo score of ≤ 2 points and no individual subscore > 1 point. Clinical response at 52 weeks was available based on partial and complete Mayo scores from VARSITY, and because these numbers were similar (vedolizumab vs adalimumab relative risk based on complete and partial Mayo scores were 1.28 and 1.22, respectively), it was deemed appropriate to proceed with the inclusion of VARSITY within the efficacy analysis based on partial Mayo score. All clinical response and remission definitions were used during the induction period (6, 8, or 10 weeks) and maintenance period (52, 54, or 60 weeks), as determined by the clinical trials [5]. To provide consistent clinical response and/or remission estimates of all studies, the network meta-analysis adjusted for differing study designs (eg, treat-through vs rerandomization) by converting maintenance clinical response and remission among induction starters for treat-through trials to maintenance response and remission among responders at the start of maintenance [5]. Wholesale acquisition cost (WAC), accessed April 8, 2021, from REDBOOK [16], was used to calculate costs of induction and maintenance regimens for labeled dosing (Table 1). WAC represents the publicly available list price for a drug and does not include rebates or discounts.

**Table 1:** Annual cost per advanced therapy regimen^a^ (WAC)

Advanced therapy	Cost, US$ [16]			NNT, remission [5]		Cost per remission, US$
	Induction period	Maintenance period	Total	Induction period	Maintenance period	At 52 weeks
Tofacitinib 10 mg PO BID^b^	9204	55,221	64,425	6.7	2.6	205,240
Tofacitinib 5 mg PO BID^b^	9204	55,221	64,425	6.7	3.4	249,417
Infliximab 5 mg/kg IV Q8W^c^	14,536	29,072	43,608	5.0	6.7	267,463
Vedolizumab 300 mg IV Q8W^d^	22,259	44,518	66,777	9.2	3.6	365,050
Golimumab 100 mg SC Q4W^e^	18,170	72,680	90,850	8.3	5.9	579,622
Adalimumab 40 mg SC Q2W^f^	17,905	71,618	89,523	17.5	6.1	750,200
Ustekinumab 90 mg SC Q8W^g^	5277	145,145	150,422	6.3	5.2	787,998

*BID* twice daily, *IV* intravenous, *NNT* numbers needed to treat, *PO* by mouth, *Q2W* every 2 weeks, *Q4W* every 4 weeks, *Q8W* every 8 weeks, *SC* subcutaneous, *US$* United States dollars, *WAC* wholesale acquisition cost

^a^Headings reflect induction- and maintenance-labeled dosing per patient per year

^b^Induction regimen: 10 mg twice daily for at least 8 weeks

^c^Induction regimen: 5 mg/kg at 0, 2, and 6 weeks

^d^Induction regimen: 300 mg at 0, 2, and 6 weeks

^e^Induction regimen: 200 mg at week 0, followed by 100 mg at 2 weeks

^f^Induction regimen: 4 injections in 1 day or 2 injections per day for 2 consecutive days

^g^Induction regimen: Single dose using weight-based dosing (260 mg for up to 55 kg, 390 mg for > 55 kg to 85 kg, 520 mg for > 85 kg)

Numbers needed to harm (NNH) for SAEs and SIs were used to estimate the healthcare costs associated with managing the respective safety-related outcomes associated with each advanced therapy. SAEs and SIs in this analysis represent SAEs and SIs that were mutually reported in the clinical trials and included in the network meta-analysis (acute hypersensitivity reaction, severe skin reactions, unspecified B-cell lymphoma, and inpatient-treated infection [including tuberculosis and opportunistic infections]).

### Model calculations

The network meta-analysis model standardized the maintenance response and remission outcomes between studies categorically as no response, response but no remission, and remission [5]. Cost per outcome (response or remission) was calculated for each advanced therapy by multiplying the NNT with the cost of the respective drug regimen. For example, a therapy that costs $50,000/year and has a 52-week NNT for a remission score of 5 would result in a cost per remission of $250,000. Five patients would have to be treated for 1 patient to achieve and maintain remission at 52 weeks. Costs of drug regimens and efficacy rates differ during the induction and maintenance periods and were calculated independently before combining periods. Patients who were treated but did not respond during the induction period incurred induction costs but did not progress to maintenance therapy on the respective drugs.

**Fig. 1:**
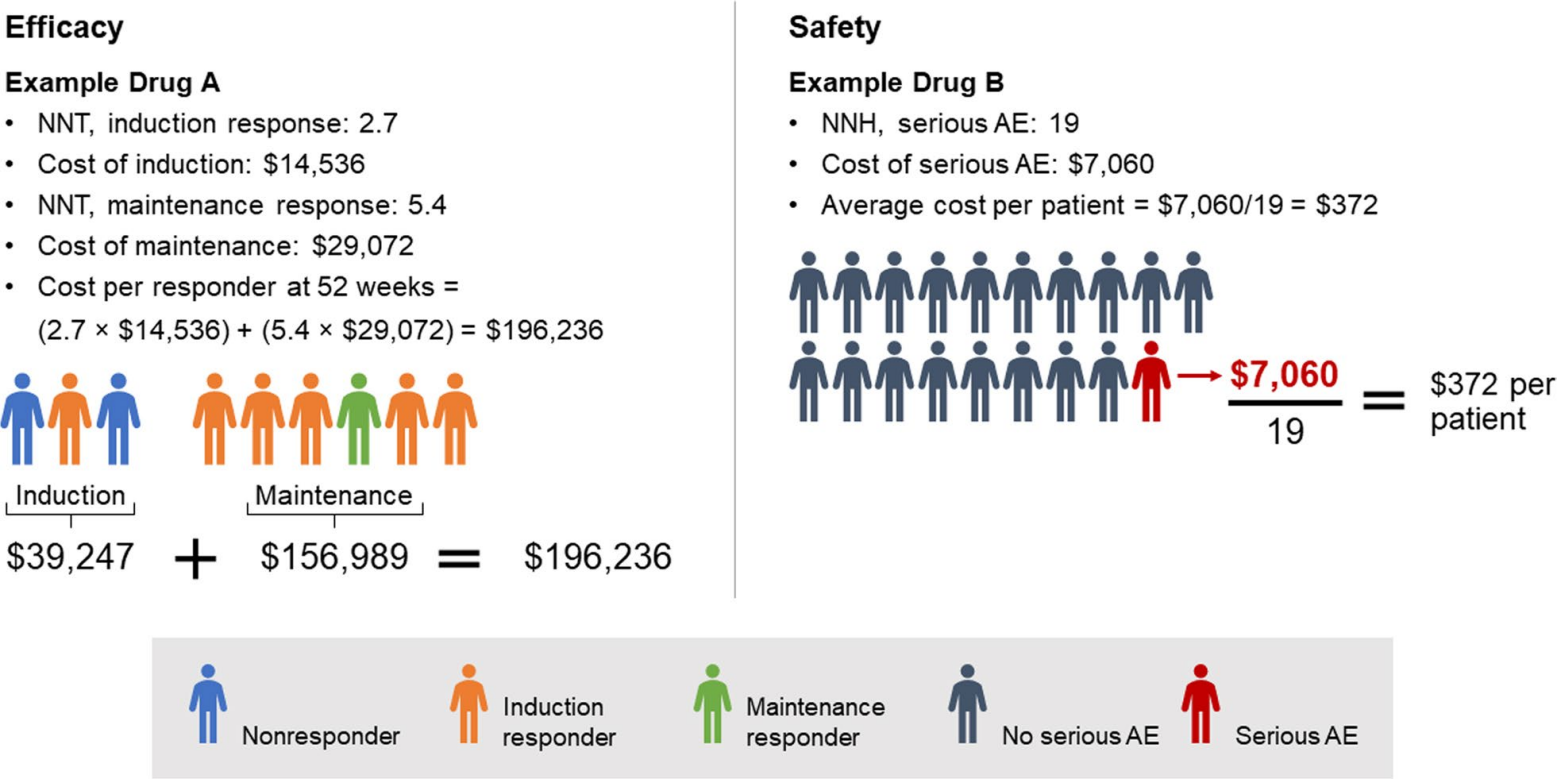
Example calculations of costs per responder and costs per SAE. *AE* adverse event, *NNH* number needed to harm, *NNT* number needed to treat, *SAE* serious AE

Costs per SAE and SI per patient were calculated for each comparator by dividing the cost of the adverse event by the advanced therapy’s NNH (eg, an inpatient’s treated infection costs $10,774 and a therapy with an NNH for serious infection score of 10 would result in an additional $1077 per patient for that therapy compared with the reference). Vedolizumab was used as the reference drug for safety-related costs because its SAE rate was numerically the lowest in the network meta-analysis, though no statistically significant differences were observed [5]. The costs for treating the respective safety outcomes were derived from the Healthcare Cost and Utilization Project [17] and represent the average reimbursable hospital costs to treat the respective AEs (eg, costs of medication, personnel, equipment, services), weighted by the risk of each respective safety outcome (Table 2). Example calculations for costs per response and costs per SAE and SI are shown in Fig. 1.

**Table 2:** Mean weighted costs for adverse events and serious infections

	Cost, US$ [18]
Serious AEs	7060
Serious infections	10,774

Serious AEs include B-cell lymphomas, acute hypersensitivity reactions, and severe skin reactions. Serious infections include inpatient-treated infections including tuberculosis and opportunistic infections. Costs were weighted based on the frequency of the respective AEs observed in the pivotal trials

*AEs* adverse events, *US$* United States dollars

### Sensitivity analyses

Two sensitivity analyses were performed to investigate the impact of dose escalation and site-of-care on cost per outcome. A systematic literature review, following the PRISMA guidelines [18], was performed in PubMed to identify published literature illustrating dose-escalation and site-of-care inputs (see Additional file 1: Fig. S1, systematic literature review search terms and PRISMA diagrams). There were 207 sources identified using search terms for “dose escalation” and 326 sources using search terms for “site-of-care.” Subsequent title, abstract, and full text review by the authors resulted in 3 appropriate sources for dose escalation and 2 appropriate sources for site-of-care. Authors reviewed the appropriate sources and collectively decided on 1 source for each of the sensitivity analyses based on the strength of the evidence and appropriateness of the endpoints investigated.

A sensitivity analysis was performed to examine dose escalation of vedolizumab, adalimumab, ustekinumab, golimumab, and infliximab [5, 19]. Dose escalation was defined as either a decrease in dosing interval or an increase in administered dose. Results revealing the prevalence of dose escalation along with the magnitude of dose escalation were used to calculate the overall increase in drug consumption for each advanced therapy. Observational evidence identified in the systematic literature review reports an increase in consumption above labeled dose of 14% for vedolizumab, 28% each for infliximab and ustekinumab, 16% for adalimumab, and 6% for golimumab. Infliximab had the highest prevalence of dose escalation and ustekinumab had the highest increase in dose (see Additional file 1: Table S1, dose escalation estimates).

An additional sensitivity analysis examined costs associated with site-of-care. The cost of IV-infused advanced therapies (vedolizumab and infliximab) are different based on the site where they are infused (physician office vs home infusion vs outpatient hospital) [20]. For example, it has been shown that costs related to outpatient hospital infusions are about 74%–84% more expensive than those for home infusions [20]. Observational evidence identified in the systematic literature review reported that the proportion of administrations that occurred in a physician’s office, at home, and in an outpatient hospital setting were 51.3%, 9.0%, and 39.7%, respectively, for vedolizumab, and 55.7%, 6.1%, and 38.2%, respectively, for infliximab [20]. The costs at each site-of-care were also reported [14] and can be found in Additional file 1: Table S2, which lists costs per infusion by site-of-care.

## Results

**Fig. 2:**
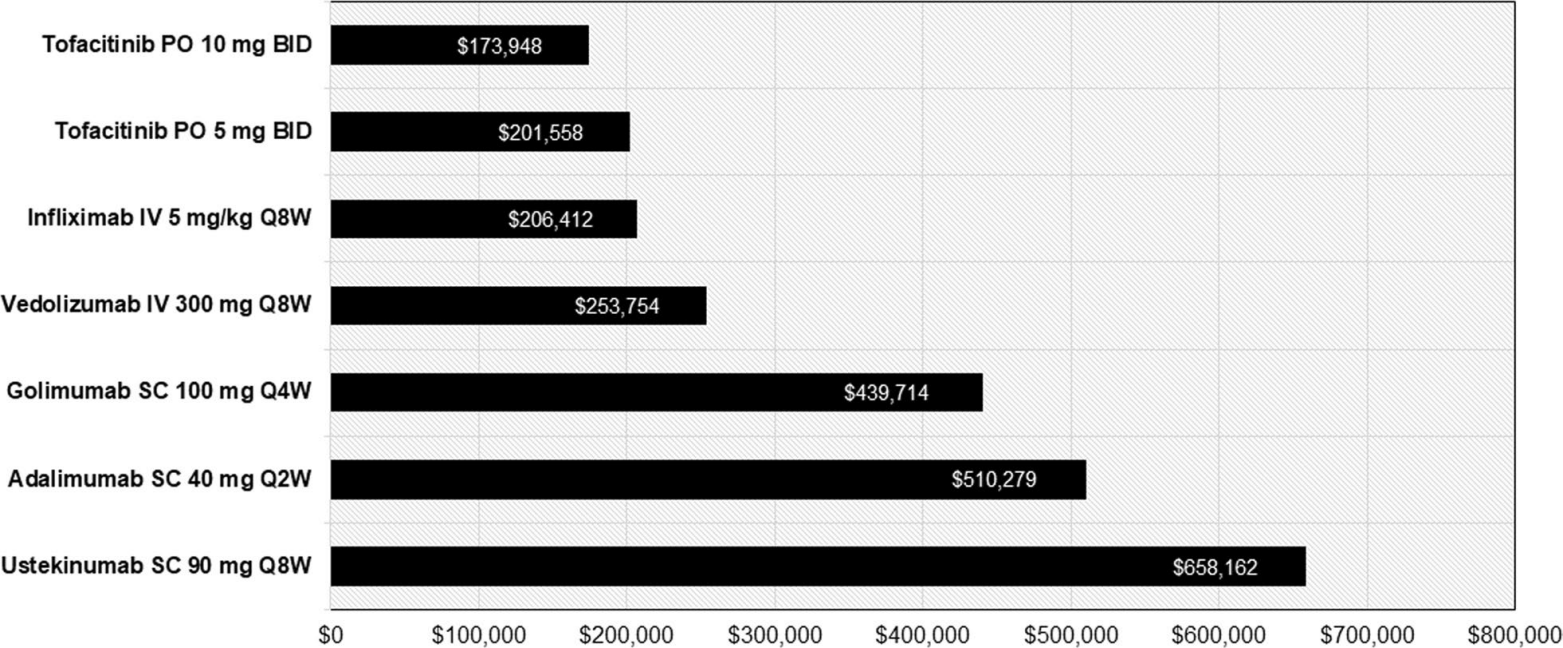
Cost per responder at 52 weeks among patients with UC treated with an advanced therapy. *BID* twice daily, *IV* intravenous, *PO* by mouth, *Q2W* every 2 weeks, *Q4W* every 4 weeks, *Q8W* every 8 weeks, *SC* subcutaneous, *UC* ulcerative colitis

Costs per response after 52 weeks of treatment were lowest for tofacitinib 10 mg and 5 mg ($173,948 and $201,558, respectively) followed by infliximab ($206,412), vedolizumab ($253,754), golimumab ($439,714), adalimumab ($510,279), and ustekinumab ($658,162) (Fig. 2).

**Fig. 3:**
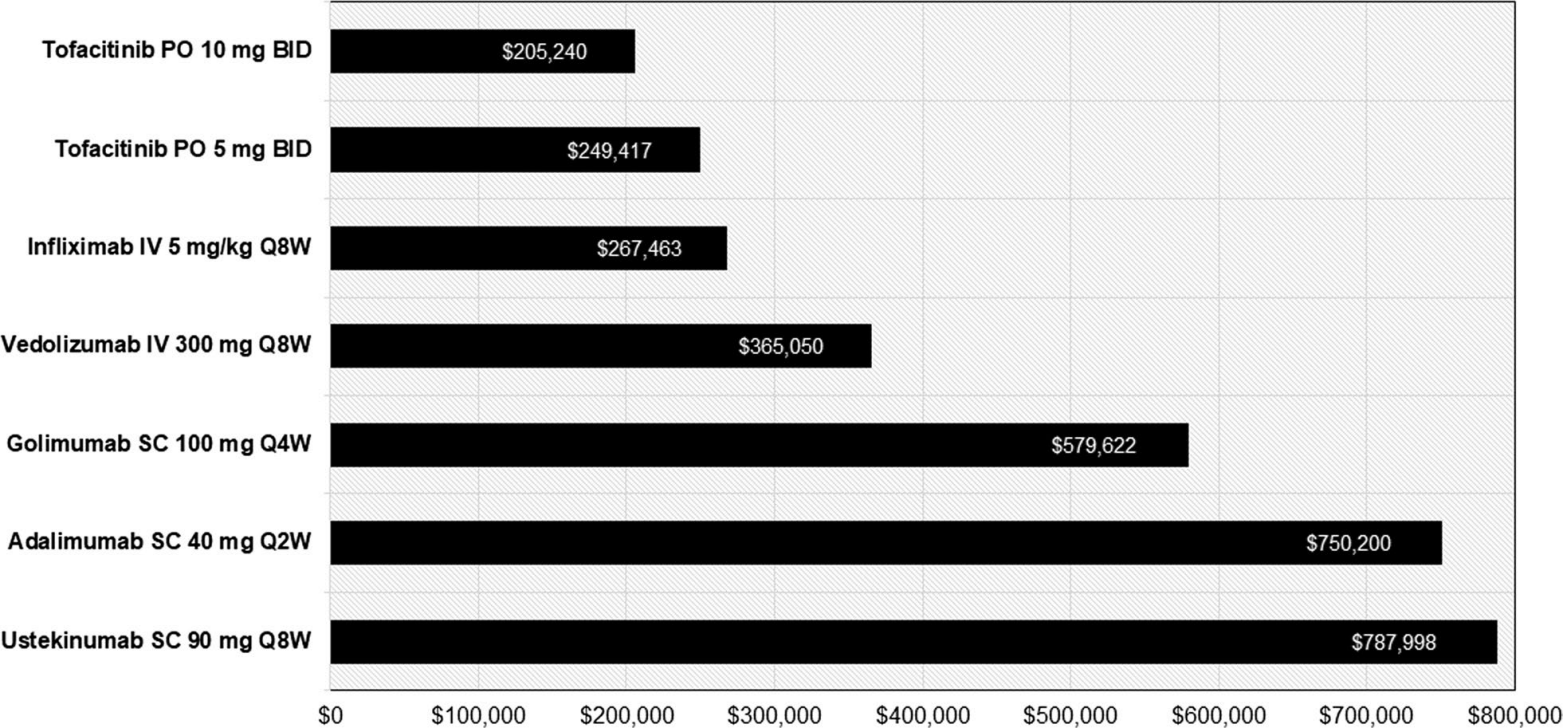
Cost per remission at 52 weeks among patients with UC treated with an advanced therapy. *BID* twice daily, *IV* intravenous, *PO* by mouth, *Q2W* every 2 weeks, *Q4W* every 4 weeks, *Q8W* every 8 weeks, *SC* subcutaneous, *UC* ulcerative colitis

Costs per remission after 52 weeks of treatment were lowest for tofacitinib 10 mg and 5 mg ($205,240 and $249,417, respectively) followed by infliximab ($267,463), vedolizumab ($365,050), golimumab ($579,622), adalimumab ($750,200), and ustekinumab ($787,998) (Fig. 3).

**Fig. 4:**
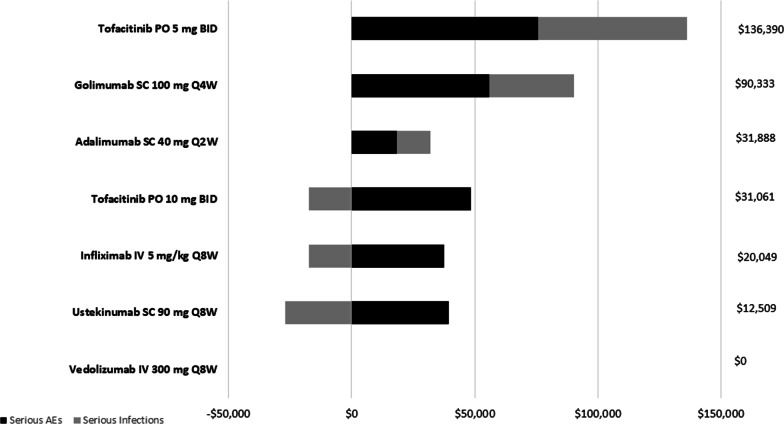
Mean cost of SAEs and SIs per 100 patients with UC treated with an advanced therapy. Dollar amounts represent the net combined costs for both SAEs and SIs. Bars to the left of $0 mark represent costs that are lower than those of the reference treatment vedolizumab. These lower costs for SIs partially offset the costs that are higher than those of the reference treatment to the right of the bar. *BID* twice daily, *IV* intravenous, *PO* by mouth, *Q2W* every 2 weeks, *Q4W* every 4 weeks, *Q8W* every 8 weeks, *SAEs* serious adverse events, *SC* subcutaneous, *SIs* serious infections, *UC* ulcerative colitis

Mean costs of combined SAEs and SIs per 100 patients were lowest for vedolizumab (set to $0 as the reference value) followed by an additional cost in reference to vedolizumab for ustekinumab ($12,509), infliximab ($20,049), tofacitinib 10 mg ($31,061), adalimumab ($31,888), golimumab ($90,333), and tofacitinib 5 mg ($136,390) (Fig. 4). Patients treated with ustekinumab, infliximab, or tofacitinib 10 mg had lower SI-related costs compared with vedolizumab, but higher overall safety costs once the costs of all SAEs were accounted for.

### Sensitivity analyses

As seen in Additional file 1: Table S1, which shows dose-escalation estimates, incorporating dose-escalation estimates for vedolizumab, infliximab, ustekinumab, adalimumab, and golimumab resulted in 14%, 28%, 28%, 16%, and 6% increases in cost, respectively. Although dose-escalation estimates and results were differential, they did not change the conclusion relative to the base case analysis; the order of lowest to highest cost per outcome remained the same (see Additional file 1: Table S2, Figs. S2 and S3).

When incorporating increased costs related to site-of-care for IV infusions, the cost per response ($319,252) and cost per remission ($459,275) after 52 weeks of treatment with vedolizumab were 28.3% higher than the base case analysis. Cost per response ($263,994) and cost per remission ($342,076) were 27.9% higher for infliximab in the site-of-care sensitivity analysis (see Table 3; Additional file 1: Fig. S4 and S5). Incorporation of increased costs associated with the site-of-care for therapies administered via infusion did not change the conclusion relative to the base case analysis; the order of lowest to highest cost per outcome remained the same.

**Table 3:** Sensitivity analysis results

	Dose escalation		Site-of-care	
	Cost per remission, US$	Increase from base case, US$	Cost per remission, US$	Increase from base case, US$
Tofacitinib 10 mg PO BID	205,240	$0	205,240	–
Tofacitinib 5 mg PO BID	249,417	$0	249,417	–
Infliximab 5 mg/kg IV Q8W	322,003	54,540	342,076	74,613
Vedolizumab 300 mg IV Q8W	387,487	22,437	459,275	94,225
Golimumab 100 mg SC Q4W	605,351	25,729	579,622	–
Adalimumab 40 mg SC Q2W	820,100	69,900	750,200	–
Ustekinumab 90 mg SC Q8W	999,329	211,331	787,998	–

*BID* twice daily, *IV* intravenous, *PO* by mouth, *Q2W* every 2 weeks, *Q4W* every 4 weeks, *Q8W* every 8 weeks, *SC* subcutaneous, *US$* United States dollars

## Discussion

This study provides valuable insights into which advanced therapies for UC provide the highest efficacy relative to their cost to treat, while also incorporating economic consequences from AEs. This evidence provides a consistent evaluation of several advanced therapies in UC, giving prescribers a robust framework to help inform treatment decisions when several options are appropriate. This evidence may be used to help contain costs in UC treatment while supporting the use of highly efficacious advanced therapies, as well as treatment positioning.

Drug acquisition costs had the largest impact on the cost per outcome results; however, differences in efficacy among the advanced therapies resulted in some having a more favorable cost per outcome than a less expensive therapy (eg, golimumab has a higher drug acquisition cost than adalimumab, but a lower cost per outcome due to higher efficacy).

Avoidance of SAEs and SIs may translate into lower healthcare costs. Costs associated with managing AEs were lowest for vedolizumab, ustekinumab, and infliximab, highlighting their favorable safety profiles from the network meta-analysis. Tofacitinib 5 mg and golimumab had substantially higher costs associated with SAEs and SIs due to higher risk observed in the network meta-analysis. Unexpectedly, tofacitinib 10 mg had lower costs on SAEs than the 5-mg dose, possibly due to the infrequency of SIs observed in clinical trials and sample size restrictions. However, tofacitinib 10 mg had higher overall rates of infections (including infections that didn’t result in hospitalization) compared with tofacitinib 5 mg in the clinical trials, consistent with dose-related AEs seen in real-world safety studies [5, 21].

Cost per outcome and cost for managing SAEs and SIs may be best interpreted as a comparative scale as opposed to an absolute number. This analysis does not include cost offsets from avoidance of UC-related complications (eg, surgery) and positive impacts on quality of life, which may not be quantitatively measurable. Therefore, the cost per outcome ratio represents the expenditures on management with advanced therapies but does not consider the savings from improved overall health. It also does not consider patients who may achieve a delayed response/remission upon dose escalation, or costs associated with treatment failure and multiple lines of treatment.

The sensitivity analyses provide context on the impacts of real-world considerations on healthcare costs. Costs for all therapies, except tofacitinib, increased substantially when applying real-world dose-escalation rates. Ustekinumab and infliximab had the highest increases in costs due to dose escalation; however, the relative order of the lowest to highest cost per outcome for all therapies remained the same. The evidence used to inform the dose-escalation inputs represented patients with IBD, including Crohn’s disease. This may have led to a small overestimation of dose escalation due to differences in dose escalation between UC and Crohn’s disease. Increases in cost due to site-of-care considerations were similar between infliximab and vedolizumab and did not result in either of the therapies having a higher cost per outcome than the SC therapies.

The results from this study are similar to those from a previous study conducted in the United Kingdom on anti–TNF-naïve patients with UC, which reported lower costs per patient with sustained response and remission at 52 weeks for vedolizumab relative to infliximab, adalimumab, and golimumab [22]. Similar results have also been observed in a Canadian study, in which golimumab had a lower cost per remission relative to adalimumab, although in contrast to the outcomes reported in this study, golimumab was associated with a lower cost per response and remission than was infliximab [23].

The incorporation of strong comparative data from the network meta-analysis, which includes 7 comparators with similar study designs and a head-to-head randomized clinical trial, is a key strength and differentiator of this study. Prior to the head-to-head (vedolizumab vs adalimumab) VARSITY study, comparative evidence was limited to indirect comparisons only. Having a stronger network of comparative evidence provides the statistical power to allow for the incorporation of more valid and reliable estimates.

The heterogeneity of the randomized clinical trials included in this analysis (ie, differences in outcome definitions, time points, and trial designs) limits the validity of the comparative efficacy outcomes and, subsequently, the overall cost per outcome results. Further details on the limitations of the NMA can be found in Jairath et al. 2021 [5].

Due to the reliance on clinical trial data, we did not include estimates elucidated outside the clinical trial setting (eg, post-marketing safety surveillance showing association of tofacitinib 10 mg with greater risk of SIs compared with 5 mg dosing) [11, 24]. In fact, because of the limited sample size and clinical trial design, tofacitinib 10 mg was associated with the second-lowest SI rate in this analysis, but this does not take into account subsequently published surveillance studies of patients with rheumatoid arthritis aged > 50 years with at least one cardiovascular risk factor that have suggested higher rates of cardiovascular events with higher doses of tofacitinib [24]. Another limitation is generalizability of cost estimates for the management of SAEs and SIs. While we used national cost averages, cost estimates for the management of SAEs vary by region. The WAC used for calculating drug acquisition costs does not reflect real-world discounts and rebates realized by the healthcare sector. Notably, biosimilars were excluded from this analysis. Given expected decreases in drug price, one may conceivably argue that market forces may ultimately reform drug pricing paradigms.

## Conclusion

In these analyses incorporating NNT and NNH derived from pivotal trials, among advanced treatment options for UC (vedolizumab, infliximab, tofacitinib, adalimumab, ustekinumab, and golimumab), tofacitinib was associated with the lowest treatment costs for obtaining clinical response and remission in patients with UC in the US, while vedolizumab offered the lowest relative costs associated with SAEs and SIs. These analyses can help inform payers, prescribers, and patients on the value of the available advanced treatment options for UC by illustrating their efficacy and safety relative to their price.

## Supplementary Information

**Additional file 1.** **Supplementary Table 2.** Costs Per Infusion by Site-of-Care. **Supplementary Figure 1.** Systematic Literature Review Search Terms and PRISMA Diagrams. **Supplementary Figure 2.** Cost Per Responder at 52 Weeks Based on a Sensitivity Analysis Incorporating Dose-Escalation Costs for Patients With UC Treated With an Advanced Therapy. **Supplementary Figure 3.** Cost Per Remitter at 52 Weeks Based on a Sensitivity Analysis Incorporating Dose Escalation Costs for Patients With UC Treated With an Advanced Therapy. **Supplementary Figure 4.** Cost Per Responder at 52 Weeks Based on a Sensitivity Analysis Incorporating Site-of-Care Costs for Patients With UC Treated With an Advanced Therapy. **Supplementary Figure 5.** Cost Per Remitter at 52 Weeks Based on a Sensitivity Analysis Incorporating Site-of-Care Costs for Patients With UC Treated With an Advanced Therapy.

## Data Availability

This model used publicly available data, combining published clinical comparative efficacy data (https://link.sgringer.com/article/10.1007/s4l669-022-0033l-9), and publicly available drug prices (https://www.micromedexsolutions.com/home/dispatch). Model calculations are described in the manuscript and all outputs are reported.

## References

[1] Shivashankar R, Tremaine WJ, Harmsen WS, Loftus EV (2017). Incidence and prevalence of Crohn's disease and ulcerative colitis in Olmsted County, Minnesota from 1970 through 2010. Clin Gastroenterol Hepatol.

[2] Lichtenstein GR, Shahabi A, Seabury SA, Lakdawalla DN, Espinosa OD, Green S (2020). Lifetime economic burden of Crohn's disease and ulcerative colitis by age at diagnosis. Clin Gastroenterol Hepatol.

[3] Park KT, Ehrlich OG, Allen JI, Meadows P, Szigethy EM, Henrichsen K (2020). The cost of inflammatory bowel disease: an initiative from the Crohn's & Colitis Foundation. Inflamm Bowel Dis.

[4] Sands BE, Peyrin-Biroulet L, Loftus EV, Danese S, Colombel J-F, Törüner M (2019). Vedolizumab versus adalimumab for moderate-to-severe ulcerative colitis. N Engl J Med.

[5] Jairath V, Chan K, Lasch K, Keeping S, Agboton C, Blake A (2021). Integrating efficacy and safety of vedolizumab compared with other advanced therapies to assess net clinical benefit of ulcerative colitis treatments: a network meta-analysis. Expert Rev Gastroenterol Hepatol.

[6] Actis GC, Pellicano R (2017). Inflammatory bowel disease: efficient remission maintenance is crucial for cost containment. World J Gastrointest Pharmacol Ther.

[7] Huoponen S, Blom M (2015). A systematic review of the cost-effectiveness of biologics for the treatment of inflammatory bowel diseases. PLoS ONE.

[8] Principi M, Labarile N, Bianchi FP, Contaldo A, Tafuri S, Lerardi E (2020). The cost of inflammatory bowel disease management matches with clinical course: a single outpatient centre analysis. Int J Environ Res Public Health.

[9] Entyvio [package insert]. Lexington (MA): Takeda Pharmaceuticals U.S.A., Inc.; 2021.

[10] Remicade [package insert]. Horsham (PA): Janssen Biotech, Inc.; 2020.

[11] Xeljanz [package insert]. New York (NY): Pfizer; 2020.

[12] Humira [package insert]. North Chicago (IL): AbbVie Inc.; 2021.

[13] Stelara [package insert]. Horsham (PA): Janssen Biotech, Inc.; 2019.

[14] Simponi [package insert]. Prescribing information. Horsham (PA): Janssen Biotech, Inc.; 2018.

[15] Jairath V, Chan K, Lasch K, Keeping S, Agboton S, Blake A (2019). Comparing the efficacy and safety of subcutaneous vedolizumab versus adalimumab for the treatment of ulcerative colitis: a network meta-analysis. Am J Gastroenterol.

[16] REDBOOK. Micomedex. Greenwood Village: Truven Health Analytics.

[17] US Department of Health & Human Services. Agency for Health Research and Quality. Healthcare cost and utilization project. https://hcupnet.ahrq.gov. Accessed 21 Aug 2021.

[18] Page M, McKenzie J, Bossuyt P, Boutron I, Hoffmann TC, Mulrow CD (2021). The PRISMA 2020 statement: an updated guideline for reporting systematic reviews. PLoS ONE.

[19] Ehrenberg R, Griffith J, Theigs C, McDonald B (2020). Dose escalation assessment among targeted immunomodulators in the management of inflammatory bowel disease. J Manag Care Spec Pharm.

[20] Talon B, Huang Z, Lissoos T, Null K (2018). Infusion of vedolizumab and infliximab in the physician office and at home was less costly compared with hospital outpatient settings. J Manag Care Spec Pharm.

[21] Sandborn WJ, Su C, Sands BE, D’Haens GR, Vermeire S, Schreiber S (2017). Tofacitinib as induction and maintenance therapy for ulcerative colitis. N Engl J Med.

[22] Jansen J, Mody R, Ursan I, Lorenzi M, Patel H, Alberton M (2015). Cost per clinical outcomes with biologics for the treatment of moderately to severely active ulcerative colitis. J Crohns Colitis.

[23] Toor K, Druyts E, Jansen JP, Thorlund K (2015). Cost per remission and cost per response with infliximab, adalimumab, and golimumab for the treatment of moderately-to-severely active ulcerative colitis. J Med Econ.

[24] US Food and Drug Administration. Initial safety trial results find increased risk of serious heart-related problems and cancer with arthritis and ulcerative colitis medicine Xeljanz, Xeljanz XR (tofacitinib). https://www.fda.gov/drugs/drug-safety-and-availability/initial-safety-trial-results-find-increased-risk-serious-heart-related-problems-and-cancer-arthritis. Accessed 26 Apr 2021.

