# Supplementary material for: Evaluating cost per remission and cost of serious adverse events of advanced therapies for ulcerative colitis
Source: BMC Gastroenterol. 2022 Dec 6;22:501. doi: 10.1186/s12876-022-02590-6 (PMC9724317; doi:10.1186/s12876-022-02590-6)
Supplement: Supplementary file 1 — Additional file 1. Supplementary Table 2. Costs Per Infusion by Site-of-Care. Supplementary Figure 1. Systematic Literature Review Search Terms and PRISMA Diagrams. Supplementary Figure 2. Cost Per Responder at 52 Weeks Based on a Sensitivity Analysis Incorporating Dose-Escalation Costs for Patients With UC Treated With an Advanced Therapy. Supplementary Figure 3. Cost Per Remitter at 52 Weeks Based on a Sensitivity Analysis Incorporating Dose Escalation Costs for Patients With UC Treated With an Advanced Therapy. Supplementary Figure 4. Cost Per Responder at 52 Weeks Based on a Sensitivity Analysis Incorporating Site-of-Care Costs for Patients With UC Treated With an Advanced Therapy. Supplementary Figure 5. Cost Per Remitter at 52 Weeks Based on a Sensitivity Analysis Incorporating Site-of-Care Costs for Patients With UC Treated With an Advanced Therapy. [file 12876_2022_2590_MOESM1_ESM.pdf]

Supplemental Material for:

Evaluating Cost Per Remission and Cost of Serious Adverse Events of Advanced Therapies for Ulcerative Colitis

BMC Gastroenterology

Jairath V, et al

Supplementary Table 1. Dose-Escalation Estimates[1]

Advanced Therapy	% of Patients Who Dose Escalated	% Increase in Drug Utilization	% Total Increase
Tofacitinib 10 mg PO BID	–	–	–
Tofacitinib 5 mg PO BID	–	–	–
Infliximab 5 mg/kg IV Q8W	39	70	28
Vedolizumab 300 mg IV Q8W	23	62	14
Golimumab 100 mg SC Q4W	14	45	6
Adalimumab 40 mg SC Q2W	28	59	16
Ustekinumab 90 mg SC Q8W	22	131	28

Proportion of patients who dose escalated and percentage increase in drug utilization represent patients with inflammatory bowel disease. BID, twice daily; IV, intravenous; PO, by mouth; Q2W, every 2 weeks; Q4W, every 4 weeks; Q8W, every 8 weeks; SC, subcutaneous.

Supplementary Table 2. Costs Per Infusion by Site-of-Care[1]

	% of Patients		Cost Per Infusion, US$	
	Vedolizumab	Infliximab	Vedolizumab	Infliximab
Physician's office	51.3	55.7	7277	4671
Home infusion	9.0	6.1	7171	5080
Outpatient hospital	39.7	38.2	12,481	8598
Total weighted cost	–	–	9335	6197
Increase in cost vs base case analysis, %	–	–	25.8	27.9

US$, United States dollars.

Supplementary Figure 1. Systematic Literature Review Search Terms and PRISMA Diagrams

Search terms for "dose escalation":
(IBD OR IBDs "inflammatory bowel disease" OR "ulcerative colitis") AND ("dose escalation" OR "dose-escalation" OR escalation) AND (biologics OR biologic OR "advanced therapy" OR "advanced therapies" OR TIM OR TIMs OR "targeted immunomodulators" OR "targeted immunomodulator")

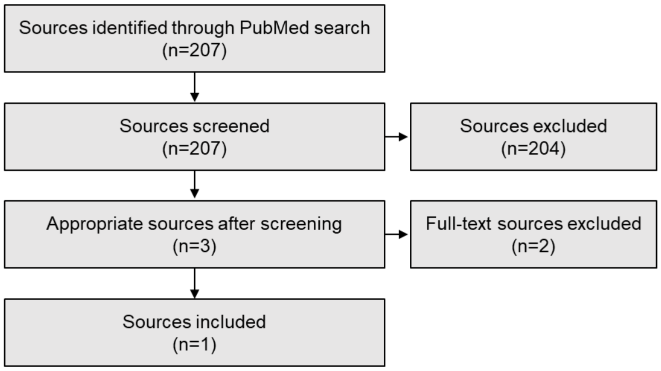

Search terms for "site-of-care":
(IBD OR IBDs "inflammatory bowel disease" OR "ulcerative colitis") AND (biologics OR biologic OR "advanced therapy" OR "advanced therapies" OR TIM OR TIMs OR "targeted immunomodulators" OR "targeted immunomodulator") AND ("site of care" OR "site of infusion" OR "infusion site" OR "infusion setting" OR "physician office" OR "home infusion" OR infusion)

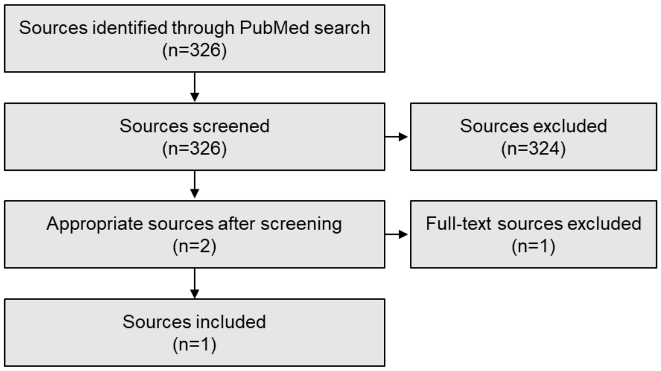

IBD, inflammatory bowel disease; TIM, targeted immunomodulator.

Search filters: published in the last 10 years, English language

Screening process: Title, abstract, and full text were reviewed by authors. For "dose escalation," sources were screened for the percentage and magnitude of dose escalation (decrease in interval or increase in dose) in all biologic therapies where dose escalation was prescribed. For "site-of-care," sources were screened for the percentage of patients who infuse at home, physician offices/infusion centers, or hospitals for intravenous-infused therapies, and the cost differences between these locations. Authors reviewed appropriate sources and collectively decided on 1 source for each of the sensitivity analyses based on the strength of the evidence and appropriateness of the endpoints investigated.

Supplementary Figure 2. Cost Per Responder at 52 Weeks Based on a Sensitivity Analysis Incorporating Dose-Escalation Costs for Patients With UC Treated With an Advanced Therapy

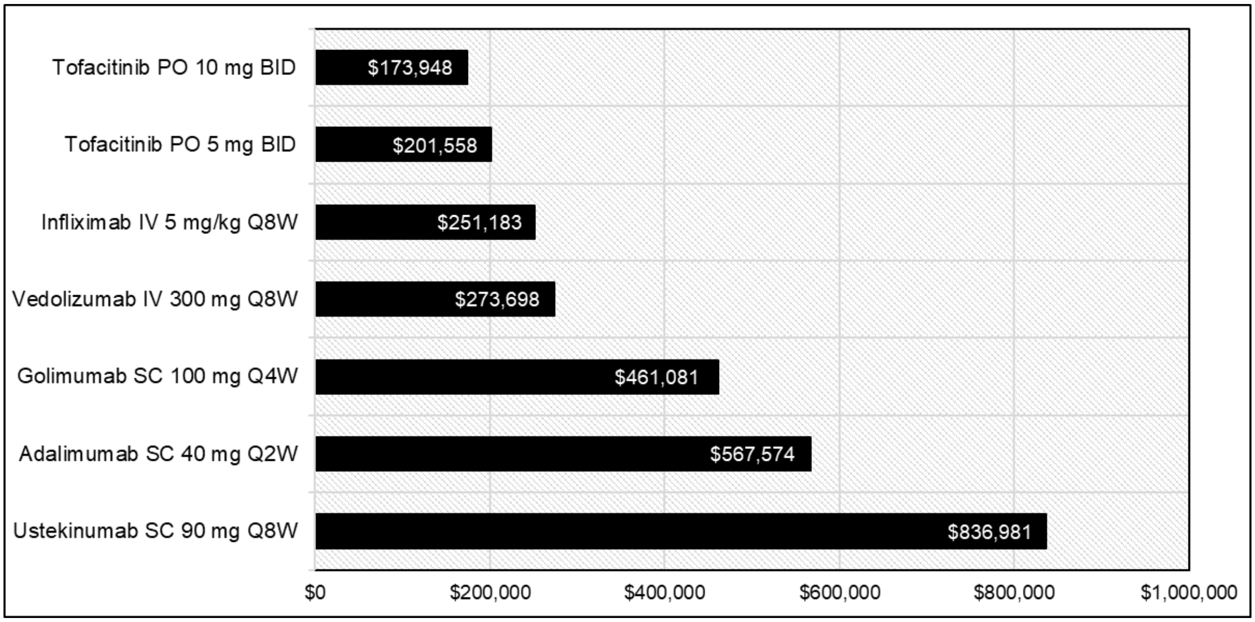

BID, twice daily; IV, intravenous; PO, by mouth; Q2W, every 2 weeks; Q4W, every 4 weeks; Q8W, every 8 weeks; SC, subcutaneous; UC, ulcerative colitis.

Supplementary Figure 3. Cost Per Remitter at 52 Weeks Based on a Sensitivity Analysis Incorporating Dose Escalation Costs for Patients With UC Treated With an Advanced Therapy

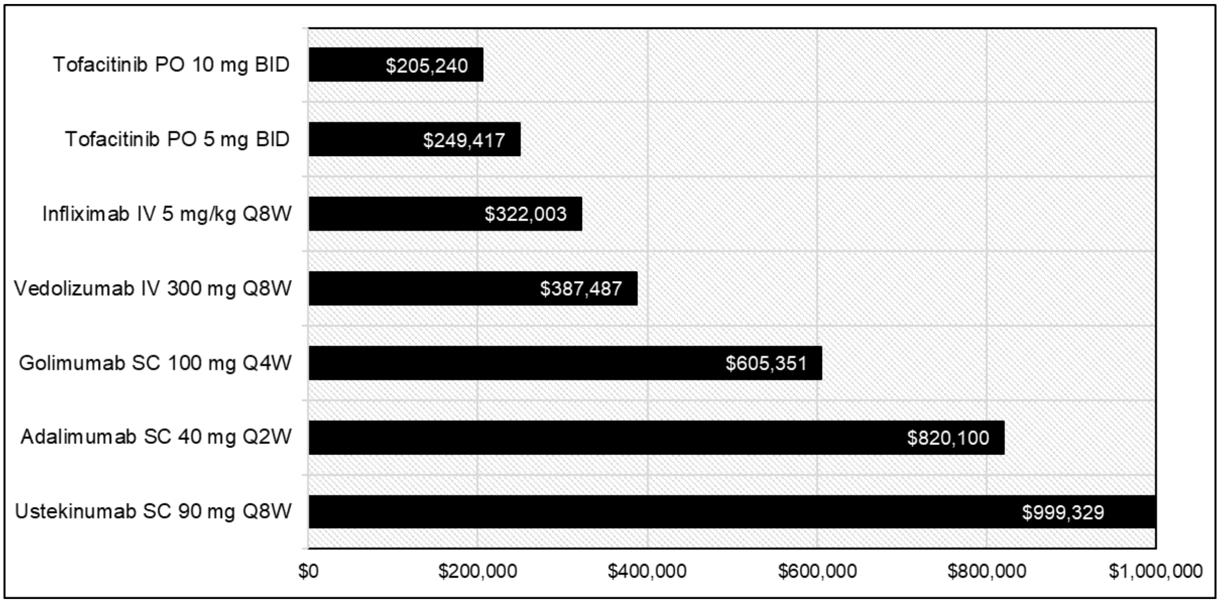

BID, twice daily; IV, intravenous; PO, by mouth; Q2W, every 2 weeks; Q4W, every 4 weeks; Q8W, every 8 weeks; SC, subcutaneous; UC, ulcerative colitis.

Supplementary Figure 4. Cost Per Responder at 52 Weeks Based on a Sensitivity Analysis Incorporating Site-of-Care Costs for Patients With UC Treated With an Advanced Therapy

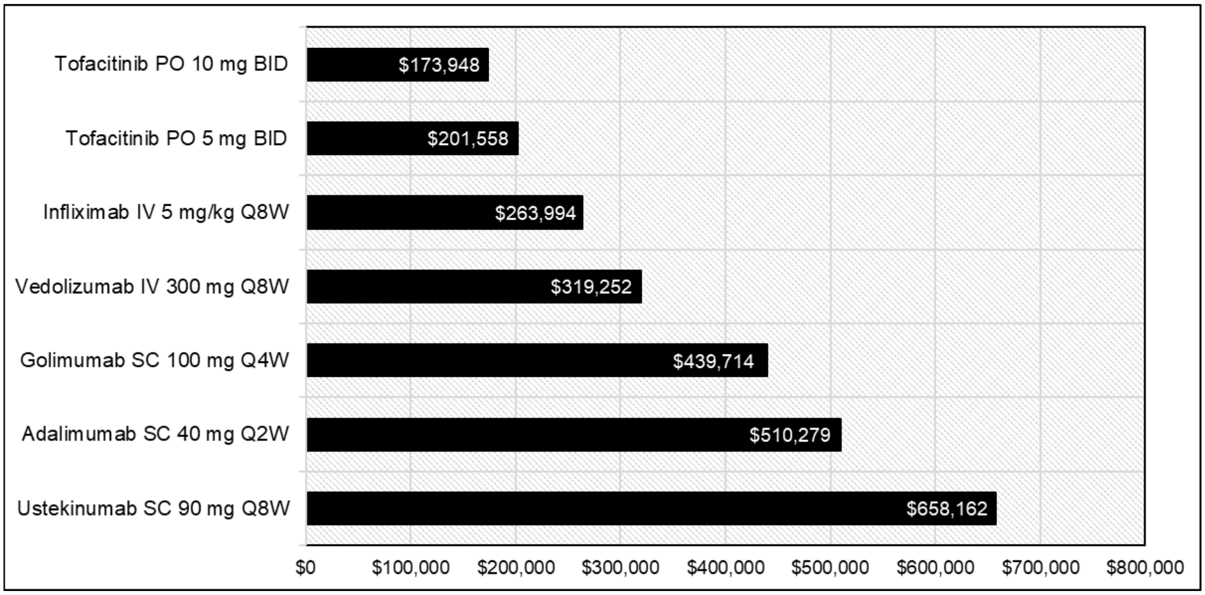

BID, twice daily; IV, intravenous; PO, by mouth; Q2W, every 2 weeks; Q4W, every 4 weeks; Q8W, every 8 weeks; SC, subcutaneous; UC, ulcerative colitis.

Supplementary Figure 5. Cost Per Remitter at 52 Weeks Based on a Sensitivity Analysis Incorporating Site-of-Care Costs for Patients With UC Treated With an Advanced Therapy

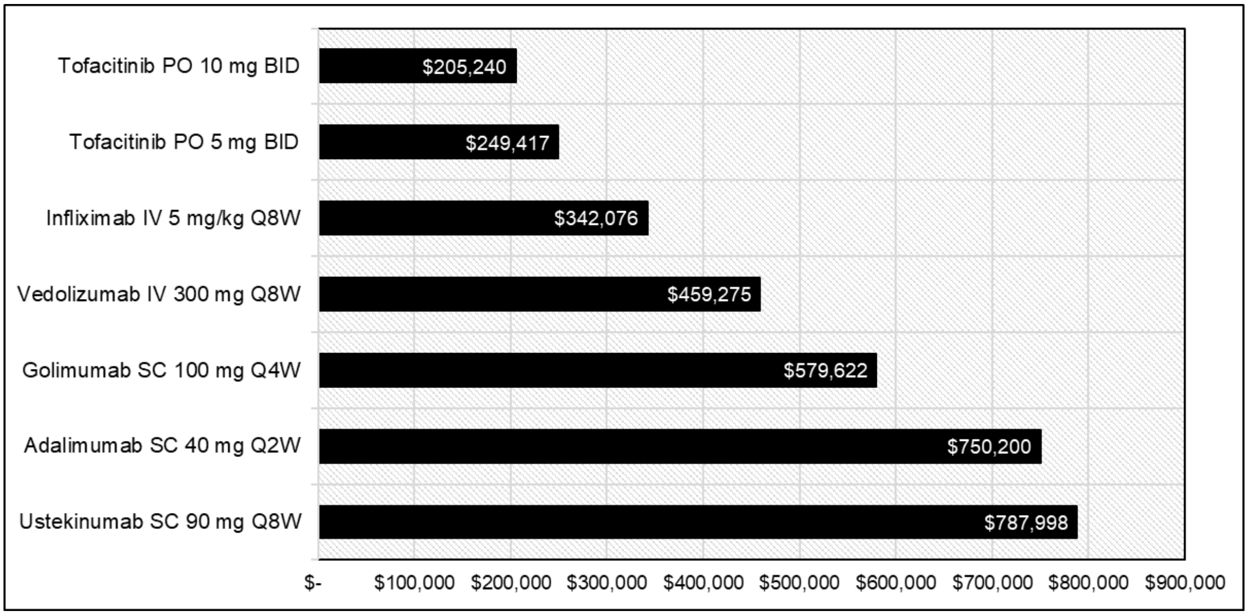

BID, twice daily; IV, intravenous; PO, by mouth; Q2W, every 2 weeks; Q4W, every 4 weeks; Q8W, every 8 weeks; SC, subcutaneous; UC, ulcerative colitis.

Reference

Ehrenberg R, Griffith J, Theigs C, McDonald B. Dose escalation assessment among targeted immunomodulators in the management of inflammatory bowel disease. J Manag Care Spec Pharm. 2020;26(6):758-765.
